# Chemical Bonding Effects and Physical Properties of Noncentrosymmetric Hexagonal Fluorocarbonates ABCO_3_F (A: K, Rb, Cs; B: Mg, Ca, Sr, Zn, Cd)

**DOI:** 10.3390/molecules27206840

**Published:** 2022-10-12

**Authors:** Yuri Zhuravlev, Victor Atuchin

**Affiliations:** 1 Department of General and Experimental Physics, Kemerovo State University, 650000 Kemerovo, Russia; 2 Laboratory of Optical Materials and Structures, Institute of Semiconductor Physics, SB RAS, 630090 Novosibirsk, Russia; 3 Department of Applied Physics, Novosibirsk State University, 630090 Novosibirsk, Russia; 4 Research and Development Department, Kemerovo State University, 650000 Kemerovo, Russia; 5 Department of Industrial Machinery Design, Novosibirsk State Technical University, 630073 Novosibirsk, Russia; 6 R&D Center “Advanced Electronic Technologies”, Tomsk State University, 634034 Tomsk, Russia

**Keywords:** DFT calculations, fluorocarbonates, crystal structure, chemical bonds, density of states, pressure, equation of state, elastic moduli, infrared absorption, Raman scattering

## Abstract

The present work applied the methods of density functional theory and the van der Waals interaction PBE + D3(BJ) on the basis of localized orbitals of the CRYSTAL17 package. It featured the effect of interactions between structural elements of fluorocarbonates ABCO_3_F (A: K, Rb, Cs; B: Mg, Ca, Sr, Zn, Cd) on their elastic and vibrational properties. The hexagonal structures proved to consist of alternating ···B-CO_3_··· and ···A-F··· layers in planes *ab*, interconnected along axis *c* by infinite chains ···F-B-F···, where cations formed polyhedra AO*_n_*F_3_ and BO*_m_*F_2_. The calculations included the band energy structure, the total and partial density of electron states, the energy and band widths of the upper *ns*- and *np*-states of alkali and alkaline-earth metals, as well as *nd*-zinc and *nd-*cadmium. For hydrostatic compression, we calculated the parameters of the *Birch*–*Murnaghan equation of state* and the linear compressibility moduli along the crystal axes and bond lines. We also defined the elastic constants of single crystals to obtain the Voigt–Reuss–Hill approximations for the elastic moduli of polycrystalline materials. The study also revealed the relationship between the elastic properties and the nature of the chemical bond. Hybrid functional B3LYP made it possible to calculate the modes of normal long-wavelength oscillations, which provided the spectra of infrared absorption and Raman scattering. Intramolecular modes *ν1* and *ν4* with one or two maxima were found to be intense, and their relative positions depended on the lengths of nonequivalent C–O bonds.

## 1. Introduction

Ultraviolet (UV) non-linear optical (NLO) crystals generate coherent UV light. As a result, they are important for laser science and technology, primarily as a clean energy source for synthesis and processing. Second-order NLO crystals operate in the UV and deep UV (DUV) ranges. They serve as frequency conversion materials in solid-state UV laser devices [1]. Most UV NLO crystals are borates with a planar ${\mathrm{BO}}_{3}^{3-}$ anion group. They possess moderate birefringence and high second-order microscopic susceptibility. Other inorganic anionic groups, e.g., ${\mathrm{NO}}_{3}^{-}$ and ${\mathrm{CO}}_{3}^{2-},$ have a π-conjugated system and a planar triangular structure. Most nitrates are soluble in water and unfit for industrial use. Single carbonate crystals are difficult to grow because they decompose at high temperatures. To obtain new NLO crystals with a low melting point, Zou et al. [2] introduced alkali fluoride into carbonates and synthesized ABCO_3_F (A: K, Rb, Cs; B: Ca, Sr) carbonates of alkaline and alkaline-earth metal fluorides. These new NLO materials demonstrated better mechanical and chemical stability. Zou et al. also measured second-harmonic generation (SHG) coefficients: they were by 1.11 (RbCaCO_3_F, CsCaCO_3_F), 3.33 (KSrCO_3_F, RbSrCO_3_F), and 3.61 (KCaCO_3_F) times greater than those of the potassium dihydrogen phosphate (KDP) crystal KH_2_PO_4_. In their other study [3], Zou et al. obtained other similar carbonates with a much higher coefficient, e.g., CsPbCO_3_F (13.4). They proved that the extremely high efficiency of SHG depended on the large *p–π* interaction between Pb^2+^ and ${\mathrm{CO}}_{3}^{2-}$ inside the layers of Pb(CO_3_). Rao et al. proved that the spin–orbit interaction affects its electronic and optical properties [4]. Xiong et al. demonstrated that a π-conjugated interaction is a common structural feature for all known DUV NLO materials with favorable properties, which makes it an important criterion for new materials [5]. An extra-large band gap and optical anisotropy are other important features of good DUV NLO crystals [6]. Therefore, the nature of the particle interaction inside the crystal and the type of chemical bond are vital for explaining the nonlinear properties and designing new NLO materials.

In [7], Zou et al. introduced two new non-centrosymmetric materials, KCdCO_3_F and RbCdCO_3_F, with extremely strong SHG responses. Their theoretical calculations confirmed that SHG efficiency depends on the interatomic interactions of *s-* and *p-*states of Cd^2+^ and π-conjugated ${\mathrm{CO}}_{3}^{2-}$ groups inside the layers of [CdCO_3_]∞. Yang et al. [8] synthesized KCdCO_3_F, RbCdCO_3_F, and KZnCO_3_F under subcritical hydrothermal conditions. Their experimental SHG response exceeded that of KDP by 4.58, 2.84, and 1.76 times. All these new compounds have wide transparency regions between UV to near-infrared (IR), which makes them promising UV NLO materials. RbMgCO_3_F is another example [9] with an SGH response of 4.0 [10]. In [11], Tran et al. studied various ABCO_3_F materials, where A was an alkali metal and B stood for an alkaline-earth metal, e.g., Zn, Cd, or Pb. Their study proved that smaller A cations and larger B cations usually increase SHG efficiency.

Structural studies are important for creating new compounds with excellent optical characteristics. Reference [10] provides an overview of NLO materials with π-conjugated ${\mathrm{CO}}_{3}^{2-}$ triangles: it revealed the link between the microscopic arrangement of ${\mathrm{CO}}_{3}^{2-}$ and macroscopic optical properties. The effect of cation radius on SHG is also featured in [12], while [2,13] focus on the crystal structures of isostructural compounds ASrCO_3_F (A: K, Rb) and KCaCO_3_F. They crystallize into a hexagonal crystal system with space group *P*-6*m*2 (No 187) with one formula unit (*Z* = 1) per unit cell. Isostructural ACdCO_3_F (A: K, Rb) and KZnCO_3_F also crystallize into a hexagonal lattice with symmetry group *P*-6*c*2 (No 188) and Z = 2 [8]. Unit cells RbBCO_3_F (B: Mg, Ca) and CsCaCO_3_F with symmetry group *P*-62*m* (No 189) contain three formula units [2,9].

Fluorocarbonate crystals are hard to synthesize and grow. As a result, all their physical properties but not optical remain experimentally understudied. Therefore, computer simulation methods usually serve this purpose. In [14], Rao et al. calculated the lattice parameters for ABCO_3_F (A: K, Rb, Cs; B: Ca, Sr) with and without the van der Waals (vdW) correction to the standard density functional theory (DFT) methods. These crystals proved to be insulators with a wide bandgap and they were all relatively harder than KDP. The same study also introduced their linear optical characteristics, e.g., dielectric function, refractive indices, birefringence, and absorption spectra. In [13], DFT calculations were applied to such mechanical and thermodynamic characteristics of ABCO_3_F (A: K, Rb; B: Ca, Sr) as entropy, Debye temperature, heat capacity, enthalpy, thermal expansion, and thermal conductivity. Another publication, [15], featured the electronic, optical, vibrational, infrared, and elastic properties of KCdCO_3_F in two structural phases. KCdCO_3_F appeared to be a direct-gap insulator with fairly flat bands below the Fermi level. However, we found no study that featured the effect of pressure on the structural properties of fluorocarbonates.

Therefore, previous studies established that fluorocarbonates have good prospects for searching for new optical materials in the ultraviolet range. They are part of a wide class of NLOs, some of which science is already familiar with and some have yet to be created. A smart search for such materials requires predictive models that can describe the macroscopic properties of a substance based on its microscopic parameters. Computer methods are a convenient tool for modeling the nature of the interaction between atoms and molecules. They can establish the effect of chemical bonding on the physical properties of crystals. If scientists know the mechanisms of chemical bonding, they can design crystal systems with desired properties. As a result, the present research objective was to analyze the features of chemical bonding in crystals of fluorocarbonates ABCO_3_F (A: K, Rb, Cs; B: Mg, Ca, Sr, Zn, Cd) and some of their main physical properties.

## 2. Calculation Method

We used the density functional theory (DFT) methods in the CRYSTAL17 software to study the structure and electronic properties of metal carbonates [16]. The software gives functions by linear combinations of localized Gaussian-type atomic orbitals and determines their exponents and coefficients from the full-electron set for carbon, oxygen, fluorine, potassium, magnesium, calcium, zinc [17], rubidium, strontium, pseudopotential cadmium [18], and cesium [19].

DFT is an exact theory, provided that the exact exchange–correlation function is available [20]. Since these are not yet available, the calculations were a generalized gradient approximation. In addition to the electron density, the approximation used data about its inhomogeneity by including the absolute value of the density gradient. Exchange potential B3 [21], the Lee–Yang–Parr correlation functional (LYP) [22], the Perdue–Burke–Ernzerhof exchange–correlation functional (PBE) [23], and the PBE functional revised for solids (PBESOL) [24] are the most popular examples. The semi-local exchange functional has a considerable drawback as it takes into account non-physical self-interaction. The exact Hartree–Fock potential can solve this problem because it provides a balance between the elimination of self-interaction and the necessity to take into account non-dynamic correlation. B3LYP was one of the first hybrid functionals to be used for this purpose. It combines the Hartree–Fock exchange (20%) with exchange functional B3 [21] and correlation functional LYP [22]. Unfortunately, such functionals presuppose adjustable parameters, which contradicts the first principles. The new functional PBE0 [25] has no adjustable parameters. We used the PBESOL0 version with an exchange–correlation functional [24] and Hartree–Fock exchange (25%). However, B3LYP still remains the best compromise between a small number of empirical adjustable parameters (three) and a wide range of highly-accurate properties. In [26,27], the authors were able to calculate the vibrational properties of alkaline-earth metal carbonates.

The reciprocal space was discretized using the Monkhorst–Pack grid [28] with 216 independent ***k***-points in the irreducible part of the Brillouin zone. The accuracy of the self-consistency procedure was ≥10^−9^ a.u. (1 a.u.= 27.21 eV).

A correct description of non-covalent interactions relies on long-range correlation effects, which DFT methods lack. To take into account the missing dispersion interaction energy, one has to increase the total energy calculated for a given density approximation [29]: E_DFT-D3_ = *E_DFT_* + *E*_disp_, where E_DFT_ is the usual self-consistent Kohn–Sham energy obtained by the functional and *E*_disp_ is the empirical dispersion correction. In the present study, we used the well-known Grimm scheme [30] and chose the dispersion correction as D3(BJ) [31].

The elastic constant tensor was calculated using the algorithm described in [32,33] as the second derivative of energy density with respect to strain pair *η* in the Voigt notation: $C_{\alpha \beta }=\frac{1}{V}{\left.\left(\frac{\partial^{2}E}{\partial \eta_{\alpha }\partial \eta_{\beta }}\right)\right|}_{\eta =0}$, where *V* is the lattice cell volume, *α*, *β* = 1–6 (1 = *xx*; 2 = *yy*; 3 = *zz*; 4 = *yz*; 5 = *xz*; 6 = *xy*). The second derivative is calculated numerically, while the first one is from the analytical gradient of the total energy.

To study the effect of pressure on the structure and electron properties, we used the equations of state (EoS) in the Birch–Murnaghan parametrization [34]. The parameters of the equation relied on the procedure described in [35]. The corresponding analytical expression was adjusted to the calculated dependence curves of the energy on the lattice cell volume *E*(*V*): $E\left(V\right)=E_{0}+\frac{9V_{0}K_{0}}{16}\left({\left(x^{-2}-1\right)}^{3}K_{1}+{\left(x^{-2}-1\right)}^{2}\left(6-4x^{-2}\right)\right)$, where $x={\left(V/V_{0}\right)}^{1/3}$,$\;K_{0}=-V{\left(\partial P/\partial V\right)}_{T}$ are isothermal modulus of volume compression, and $K_{1}={\left(\partial K/\partial P\right)}_{T}$ is its first derivative with respect to pressure at *x* = 1. *E*_0_, *V*_0_, *K*_0_, and *K*_1_ are the four EoS parameters, where *V*_0_ is the volume at the energy minimum, *E*_0_ defines the depth of the curve *E*(*V*), and *K*_0_ and *K*_1_ define its shape. Knowing these parameters, we could easily calculate the pressure as $P\left(V\right)=\frac{3K_{0}}{2}\left(x^{-7}-x^{-5}\right)\left(1+\frac{3}{4}\left(K_{1}-4\right)\left(x^{-2}-1\right)\right)$.

The harmonic frequencies of phonons at point Г (***k*** = 0, the center of the first Brillouin zone) were obtained by diagonalizing the mass-weighted matrix of the second derivatives of energy with respect to atomic displacements *u* [36]: $W_{ai,bj}^{H}=\frac{H_{ai,bj}^{0}}{\sqrt{M_{a}M_{b}}},H_{ai,bj}^{0}=\left(\frac{\partial^{2}E}{\partial u_{ai}^{0}\partial u_{bj}^{0}}\right)$, where atoms *a* and *b* that weigh *M_a_* and *M_b_* shift in the lattice cell (index 0) along Cartesian directions *i* and *j* from the equilibrium state, respectively.

The first-order derivatives were calculated analytically, while the second-order derivatives were obtained numerically. The IR absorption intensity was calculated using the Born effective charge tensor, which is the dynamic feature of a displaced atom and characterizes the change in its electronic configuration. The Stokes line intensity of phonon mode *Q_p_*, which was active as a result of the polarizability tensor α*_ii_*, was proportionate to $I_{ii}^{p}\infty {\left(\partial \alpha_{ii}/\partial Q_{p}\right)}^{2}$. The relative peak intensities were calculated analytically by using the scheme published in [37].

To characterize the correctness of linear dependencies between different quantities, we used the correlation coefficient calculated by the following formula:

$$D=\sqrt{\sum_{i=1}^{N}{\left(y_{i}^{fit}-\bar{y^{fit}}\right)}^{2}/\sum_{i=1}^{N}{\left(y_{i}^{data}-\bar{y^{data}}\right)}^{2}},$$

where $\bar{y}=\frac{1}{N}\sum_{i=1}^{N}y_{i}\mathrm{is}\;\mathrm{mean}\;\mathrm{value}$; *data* is the reference value; *fit* is the interpolated value of the corresponding quantity; and *N* is their number. The interpolation factor was given in brackets after the corresponding formula and it was omitted if its value was 1.00.

## 3. Results

### 3.1. Crystal Structure

Based on their crystal structure, all the fluorocarbonates in this research were divided into three groups: group I—KCaCO_3_F, KSrCO_3_F, RbSrCO_3_F (space group *P*-6*m*2); group II—KZnCO_3_F, KCdCO_3_F, RbCdCO_3_F (*P*-6*c*2); group III—RbMgCO_3_F, RbCaCO_3_F, CsCaCO_3_F (*P*-62*m*). Each group clearly demonstrates the roles of its alkali metal, alkaline-earth metal, or the one that contained a completely full *nd*^10^ shell in addition to the full *ns*^2^ shell.

Atoms of crystal KSrCO_3_F are layered as follows: strontium (Sr), carbon (C), and oxygen (O) are located in plane *z* = *c*/2, whereas potassium (K) and fluorine (F) atoms are in plane *z* = 0. Here and below, coordinate *z* is chosen along axes *c, x,* and *y* in plane *ab*. Atomic position coordinates are as follows: K(0,0,0), Sr(2/3,1/3,1/2), C(1/3,2/3,1/2), F(2/3,1/3,0), and O(-2*x*_O_,-*x*_O_,1/2) in lattice vectors *a* = *b*, *c*. Thus, the independent parameters of the lattice were determined by optimizing its geometry and involved three quantities: *a*, *c,* and *x*_O_. Table 1 shows the independent lattice parameters calculated using PBE + D3 and B3LYP, as well as those measured experimentally in [2,8,9]. They make it easy to calculate interatomic distances.

**Figure 1:**
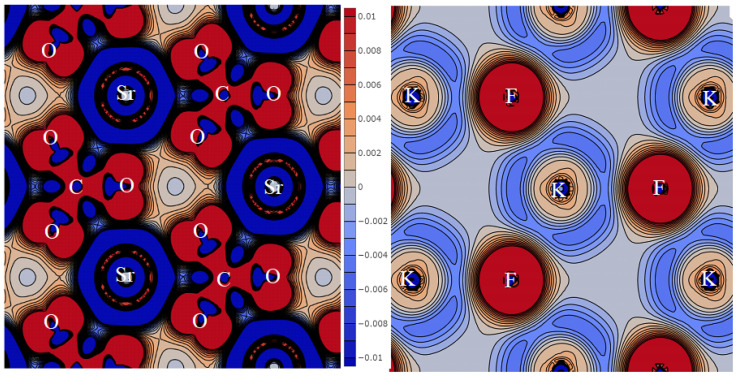
Distribution of strain density in plane *xy*, *z* = *c*/2 (**left**) and *z* = 0 (**right**) in the primitive cell of KSrCO_3_F. The letters in the figure represent the symbols of the atoms.

Figure 1 shows the deformation density distribution Δρ for KSrCO_3_F obtained by subtracting the density of non-interacting atoms from the crystal density. It illustrates the redistribution of the electronic charge during the chemical bond formation. Positive Δρ areas are red, while the areas of negative values are blue. Density values here and below are given in atomic units.

Each Sr atom is surrounded by six oxygen atoms separated by 2.622 Å. All distances are given for functional PBE + D3, and Table 1 provides them for B3LYP. In Figure 1 (left), the electron charge flows out of the inner atomic regions and flows into the outer ones. In the case of Sr, the charge follows in such a way that the interaction results in a positively charged ion +1.82 |*e*|, where *e* is the electron charge. The ion charge was calculated according to the Mulliken scheme, which splits it between atoms, which is not entirely true. The charge flows out from the inner regions of the carbon and oxygen atoms. Then, it flows into the region between them and behind the oxygen nuclei symmetrically to bond line C–O (1.308 Å). As a result, carbon acquires a positive charge of +0.39 |*e*|, while oxygen acquires a negative charge of −0.70 |*e*|. The overlap population of electron clouds on bond line C-O (*P*_C-O_) is 0.539 *e*, which indicates that they develop a strong covalent chemical bond and a stable carbonate ion. The excess charge behind the oxygen nuclei in plane *ab*, *z* = c/2 produces a stable SrCO_3_ structure. All carbonate groups line up in parallel, which gives the maximal contribution to the macroscopic SHG effect [2].

In plane *ab*, *z* = 0 (Figure 1, right), each potassium atom is surrounded by three fluorine atoms at 3.018 Å. The electronic charge flows out of the potassium atom so that its ionic charge is +0.75 |*e*|. It then flows onto fluorine with its ionic charge of −0.84 |*e*|. Fluorine has a greater affinity for the electron and attracts electronic charges from both cations. Since carbonate ion has three oxygen atoms, it has a higher negative charge. The overlap population is *P*_K-F_ = 0.019 *e*, so bond K-F is an ionic chemical. The entire layer acquires an excess negative charge of −0.09 |*e*|, which provides additional electrostatic adhesion of layers ···Sr-CO_3_··· and ···K-F··· to each other.

**Figure 2:**
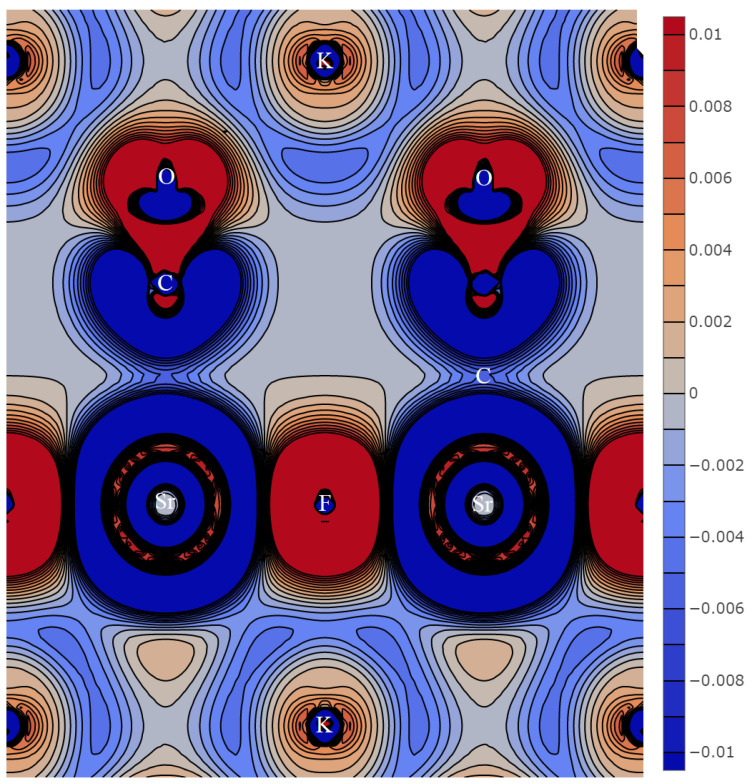
Deformation density distribution in plane *xz* (*y* = *a/2*) in the primitive cell of KSrCO_3_F.

Figure 2 shows the distribution of the deformation density in plane *xz* (*y = a*/2) in the primitive cell of KSrCO_3_F. It has strontium and fluorine atoms, as well as closely spaced oxygen and potassium atoms, so that the layers of Sr, C, and O alternate with those of K and F. The fluorine atoms are located above and below the strontium atoms at 2.354 Å. The overlap population for Sr-F is 0.003 *e*, which is even less than Sr-O at 0.008 *e* with a larger distance. Thus, the resulting ionic chemical bond provides infinite ···F-Sr-F··· chains. Each potassium atom is surrounded by six oxygen atoms at 2.909 Å. As maxima Δρ are located behind the oxygen nuclei, the overlap population on line K-O is 0.018 *e*. Thus, the nearest environment of negatively charged ions is polyhedra KO_6_F_3_ for potassium and SrO_6_F_2_ for strontium. According to [38], if the coordination number *N*_OF_ is nine, the radius of the potassium ion R_A_ equals 1.55 Å, and for a strontium ion with *N*_OF_ = 8, *R*_B_ equals 1.26 Å. As a result, the average radius of cation *R*_C_ = (*R*_A_ + *R*_B_)/2 is 1.405 Å. This value allowed us to establish all ordinary patterns in this research.

When the alkali metal cation Rb replaces K in isostructural RbSrCO_3_F, the replacement triggers no fundamental change in the parameters of the crystal structure. Constants *a* and *c* increase, as do all interatomic distances, but the quantitative crystallographic environment remains the same. The cationic charge of strontium changes insignificantly and reaches +1.81 |*e*|. For the rubidium ion, the charge is +0.84 |*e*| larger than for potassium, and its cationic radius is 1.63 Å. In KCaCO_3_F, the calcium ion radius is 1.12 Å smaller than that of strontium. Therefore, lattice constants *a* and *c* and the interatomic distances are also smaller than in other crystals. The charge of the calcium ion is much less than that of strontium and equals +1.45 |*e*|, while the charge of potassium reaches +0.76 |*e*|. Due to the smaller calcium radius, the overlap populations of Ca-O and Ca-F increase by 0.049 and 0.036 *e*, respectively. Unlike KSrCO_3_F, the charge of the Ca-CO_3_ layer is negative and equals −0.024 |*e*|.

The average radius of cation *R*_C_ in the series of KCaCO_3_F, KSrCO_3_F, and RbSrCO_3_F increases as 1.335, 1.405, and 1.445 Å, respectively. They have a stable linear dependency on the geometric parameters of the lattice. Lattice constant *a* increases as *a*(Å) = 2.815 + 1.713·*R*_C_, and so do the interatomic distances: *R*_A-O_(Å) = 0.405 + 1.778·*R*_C_, *R*_B-O_(Å) = 1.371 + 0.888·*R*_C_. The change in the distances triggers the change in the parameters of the chemical bond. As *R*_C_ increases, the charges of CO_3_ anions (−1.48, −1.71, −1.78 |*e*|) and fluorine anions (−0.73, −0.84, −0.87 |*e*|) also increase. The overlap population of bond C–O also goes up (0.516, 0.539, 0.551 *e*), which means that the covalent chemical bond in the carbonate group also increases.

Zinc and cadmium atoms have completely full 3*d*^10^ and 4*d*^10^ electron shells, which are energetically close to the upper full 4*s*^2^ shell. Therefore, *d-*electrons participate in the hybridization of electron states. As a result, fluorocabonates with zinc and cadmium have different properties.

Lattice cell KCdCO_3_F contains two formula units. Unlike group I, lattice constant *c* is twice as large, and its alternating layers are more numerous. The coordinates of nonequivalent atoms in units *a* and *c* are as follows: K(2/3,1/3,1/2), Cd(0,0,1/4), C(1/3,2/3,1/4), F(0,0,1/2), and O(*x*_O_,*y*_O_,1/4), i.e., a total of four independent quantities (see Table 1 for their values).

**Figure 3:**
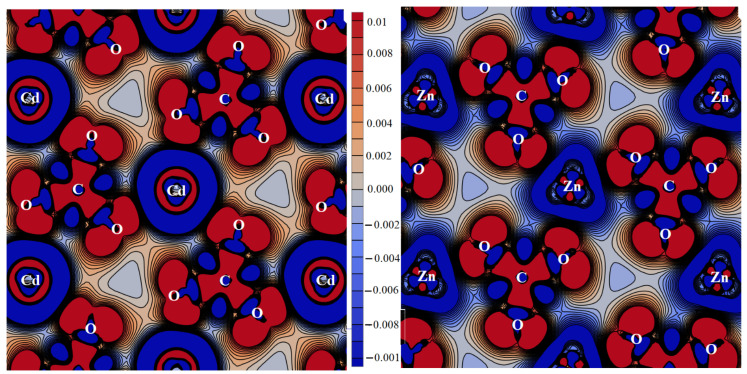
Deformation density distribution in plane *ab*: KCdCO_3_F (**left**) and KZnCO_3_F (**right**).

The cadmium atom is surrounded by three carbonate groups in plane *z* = *c*/4 (Figure 3, left). Unlike KSrCO_3_F, they are deployed in such a way that only three oxygen atoms are located at 2.238 Å, and three others are at a greater distance of 2.882 Å. This rotation leads to the fact that carbonate groups in the neighboring planes separated by *c*/2 are parallel to each other and have the same direction. These planes are connected through infinite chains ···F-Cd-F···, the shortest distance being 2.219 Å. Thus, cadmium cation forms polyhedra CdO_6_F2. For coordination number *N*_OF_ = 8, cadmium ion radius is 1.1 Å [38]. The calculated cadmium ion charge is much lower than those calculated for Ca or Sr and equals +1.07 |*e*|, which also leads to a lower charge of the carbonate group, which equals −1.23 |*e*|. The small cadmium radius is accompanied by relatively small Cd-O and Cd-F distances. As a result, they have a larger electron shell overlap population of 0.089 and 0.073 *e*, respectively, than other cations. It is only 0.016 *e* for three oxygen atoms separated by large distances.

Each potassium atom in KCdCO_3_F is surrounded by six oxygen atoms at 2.804 Å and three fluorine atoms at 2.946 Å. Thus, the radius of its ion is 1.55 Å, and its charge is +0.78 |*e*|. The charge of fluorine atoms in the same plane is −0.62 |*e*| and the total charge is much greater than in group I.

When zinc replaces cadmium in KZnCO_3_F, it decreases the lattice constants and all interatomic distances, including K-O (2.733 Å). Potassium atom charge falls down to +0.75 |*e*|, while that of fluorine rises as high as −0.68 |*e*|. The crystalline environment of the zinc atom differs from that of cadmium in KCdCO_3_F. It is surrounded by three oxygen atoms at 2.002 Å, two fluorine atoms, and three carbons, while three more oxygen atoms come last at 3.028 Å. This pattern occurs because the carbonate groups change their direction axis relative to the cations (Figure 3, right). Zinc ion charge is +1.24 |*e*| and its radius for *N*_OF_ = 8 is 0.90 Å. Such a small radius has a considerable overlap population of 0.112 and 0.066 *e* for Zn-O and Zn-F, respectively. Thus, the proportion of the covalent component of the chemical bond in KZnCO_3_F is much higher than in other fluorocarbonates.

When rubidium replaces potassium, the resulting RbCdCO_3_F experiences an increase in all interatomic distances: Rb-O rises to 2.898 Å and Rb-F increases to 3.009 Å. As a result, the rubidium ion radius charged +0.87 |*e*| is 1.66 Å. Fluorine shares the same plane with a charge of −0.64 |*e*| and the total positive charge is very significant. The environment of cadmium is the same as that of zinc. Each cadmium ion with a charge of +1.06 |*e*| is surrounded by the three nearest oxygen atoms at 2.221 Å followed by two fluorine atoms and carbon, while three oxygen atoms come last at 3.018 Å. The radius of the cadmium ion is small (1.1 Å), which provides a significant overlap of electron shells with oxygen at 0.089 *e* and fluorine at 0.073 *e*, as well as a high share of the covalent component in the chemical bond.

Group II demonstrates the linear dependency of the crystal parameters on the average cation radius, which increases as 1.225, 1325, and 1.365 Å in the series of KZnCO_3_F, KCdCO_3_F, and RbCdCO_3_F. For constant *c*(Å) = 1.918 + 5.234·*R*_C_ and distances *R*_C-O_(Å) = 1.274 + 0.022·*R*_C_ (0.99), the increasing bond length increases the population of overlap *P*_C-O_, which is 0.483, 0.493, and 0.506 *e* in this series.

In RbCaCO_3_F, atoms occupy the following positions: Rb(0,-*x*_Rb_,0), Ca(-*x*_Ca_,-*x*_Ca_,1/2), carbon C1 (1/3,2/3,1/2), C2(0,0,1/2), C1(1/3,2/3,1/2), C2(0,0,1/2), F(-*x*_F_,-*x*_F_,0), O1(*x*_O1_,-*y*_O1_,1/2), and O2(-*x*_O2_,-*x*_O2_,1/2) (see Table 1 for their values). This compound has three carbonate groups. Two C1O_3_ groups occupy equivalent positions, their C1-O1 distances being 1.308 Å. In the third C2O_3_ group, the distance of C2-O2 is 1.302 Å. The rubidium atom is surrounded by four O1 oxygen atoms at 3.015 Å, one fluorine atom at 3.117 Å, four more O2 oxygen atoms at 3.149 Å, and, finally, two more fluorine atoms at 3.155 Å. In polyhedron RbO_8_F_3_, the rubidium ion charge is +0.86 |*e*| and its radius is 1.69 Å [38]. The charge of the entire plane per one formula unit is +0.11 |*e*|.

**Figure 4:**
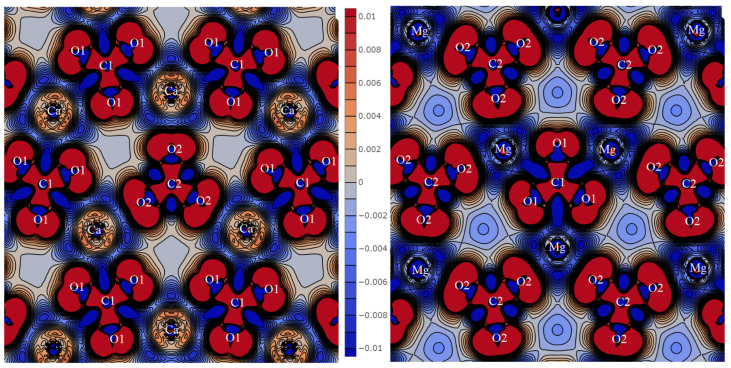
Deformation density distribution in plane *ab*: RbCaCO_3_F (**left**) and RbMgCO_3_F (**right**).

The calcium atom is surrounded by two fluorine atoms at 2.223 Å, one O2 oxygen atom at 2.275 Å, and two O1 at 2.458 Å and 2.488 Å from two C1O_3_ (Figure 4, left). In CaO_5_F2, calcium ion charge is +1.45 |*e*|, and its radius is 1.06 Å. The surrounding carbonate groups C1O_3_ and C2O_3_ with distances of 1.308 and 1.302 Å are charged as −1.54 |*e*| and −1.59 |*e*|, respectively. For the deformation density in plane *ab*, carbonate groups C1O_3_ point in the same direction (Figure 4), while groups C2O_3_ point in the opposite direction (see the center of Figure 4). Group III fluorocarbonates differ from the others.

CsCaCO_3_F maintains the same cationic environment. The cesium ion radius is 1.85 Å in CsO8F_3_ and its charge is +0.66 |*e*|. Fluorine is charged at −0.71 |*e*|, which is also lower than in a rubidium crystal. Calcium also forms pentagonal bipyramids with an ion charge of +1.48 |*e*| and a radius of 1.06 Å. The alignment of four O1 and one O2 equatorial atoms leads to an asymmetric alignment of three carbonate groups: two groups C1O_3_ with a distance for C1-O1 of 1.307 Å and a charge of −1.44 |*e*| are parallel to each other and share the edges with CaO_5_F_2_, while the third C2O_3_ group has a distance of 1.304 Å and a charge of −1.54 |*e*| and is antiparallel to the rest.

The magnesium ion in RbMgCO_3_F is the smallest of all the alkaline-earth metals in this research, so its environment differs from the other group III crystals. The rubidium atom is surrounded by ten oxygen atoms as follows: two O2 at 2.936 Å, four O1 at 3.086 Å, and four O1 at 3.162 Å. One fluorine atom is located at 3.060 and two are located at 3.106 Å. The rubidium ion in RbO_10_F_3_ has a charge of +0.85 |*e*| and a radius of 1.83 Å. The fluorine charge of −0.72 |*e*| is responsible for the excess charge of the entire plane. The magnesium atom also has a MgO_4_F_2_ environment, which is different from other alkaline-earth ions (Figure 4, right): the magnesium atom is surrounded by two fluorine atoms at 1.966 Å, two oxygen atoms O1 at 1.989 Å, and two oxygen atoms O2 at 2.173 Å from two C2O_3_. In this environment, the magnesium charge is +1.37 |*e*| and its radius is 0.72 Å. This change is associated with the reversal of carbonate groups, which polarizes the small magnesium cation. *R*_C1-O1_ = 1.298 Å in two ions C1O_3_ is less than 1.303 Å in one C2O_3_, and the charge is −1.51 |*e*| greater than −1.47 |*e*|. These peculiarities manifest in the elastic and vibrational properties of group III fluorocarbonates. Due to its small radius, the overlap population of Mg-O at 0.095 *e* and Mg-F at 0.081 *e* is significantly higher than for Ca-O at 0.04 *e*.

Like other groups, the average cation radius in the series of RbMgCO_3_F, RbCaCO_3_F, and CsCaCO_3_F is as high as 1.275, 1.375, and 1.455 Å, respectively. Crystallographic parameters *a*(Å) = 6.90 + 1.671·*R*_C_ and *R*_A-O_(Å) = 1.880 + 0.828·*R*_C_ demonstrate a linear dependence.

We compared the experimental crystallographic data for fluorocarbonates of group I [2], group II [8], RbMgCO_3_F [9], RbCaCO_3_F, and CsCaCO_3_F [2]. The mean-square deviation for eight structural parameters *VR*: *a*, *c*, *V*, *R*_A-O_, *R*_A-F_, *R*_B-O_, *R*_B-F_, and *R*_C-O_ (mean for group III) is 0.42% for PBE + D3 functional, 1.53% for PBE, and 1.26% for B3LYP for nine fluorocarbonates. PBE + D3 gives a minimal deviation of 0.35% for lattice constant *a*, 0.3% for constant *c*, 0.58% for the shortest distances between cations A and oxygen, 0.36% for cations B and oxygen, and 0.79% for hybrid functional B3LYP for C-O. PBE + D3 decreases constant *a* in KSrCO_3_F (0.6%), RbSrCO_3_F (0.25%), KZnCO_3_F (0.3%), and KCdCO_3_F (0.6%). It decreases constant *c* in KZnCO_3_F (0.4%), RbCdCO_3_F (0.2%), RbMgCO_3_F (0.2 %), and RbCaCO_3_F (0.04%). Functional PBE in all fluorocarbonates increases both constant *a* and constant *c*. Functional B3LYP leads to a similar result, except for KZnCO_3_F and RbCdCO_3_F, where constant *c* is lower by 0.1%. Thus, van der Waals interactions provide an agreement between the calculated and experimental parameters of the crystal structure in fluorocarbonates ABCO_3_F.

All fluorocarbonates demonstrate a simple linear relationship between the structural parameters *VR* calculated by different functionals. For KCaCO_3_F, it is *VR*_PBE_ = −0.052 + 1.025·*VR*_PBE + D3_. For volume *V*, the value calculated by functional PBE + D3 is 100.794 Å3, while the value obtained by the interpolation formula for PBE is 103.279 Å3, its precise value being 103.281 Å3. For functional B3LYP, the formula is *VR*_B3LYP_ = −0.047 + 1.022·*VR*_PBE + D3_. On the contrary, gradient functional PBESOL and hybrid functional PBESOL0 lead to decreased lattice constants and actual interatomic distances: *VR*_PBESOL_ = 0.038 + 0.982·*VR*_PBE + D3_, *VR*_PBESOL0_ = 0.057 + 0.972·*VR*_PBE + D_. Their mean-square deviations are 0.7 and 1.0%, which exceeds those for PBE + D3 by 0.45%.

**Figure 5:**
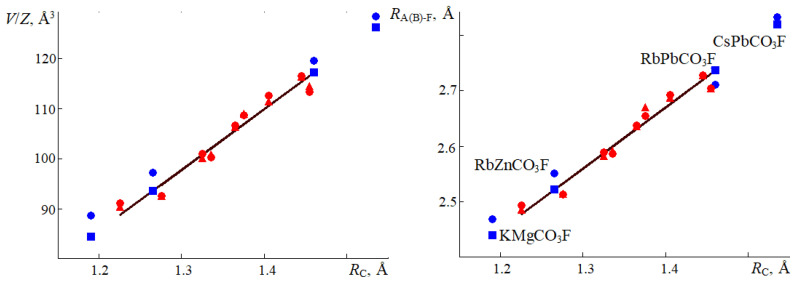
Cell volumes were calculated using functional PBE + D3 (red triangles) per one formula unit *V/Z* (**left**), the average distance between cations and fluorine atoms *R*_A(B)-F_ (**right**); cell volumes were measured experimentally [2,8,9] (red circles). The solid black line designates the interpolated linear dependence on the average cation radius *R*_C_. The blue color marks predicted (squares) and experimental (circles) values for fluorocarbonates KMgCO_3_F, RbZnCO_3_F, RBPbCO_3_F, and CsPbCO_3_F.

All fluorocarbonates demonstrated a stable linear dependence. The lattice cell volume per formula unit depends on the average cation radius *V*/*Z*(Å^3^) = −59.75 + 121.16·*R*_C_ (0.99), the average distance from cations A and B to the nearest oxygen (*R*_A–O_ + *R*_B-O_)/2(Å) = 0.253 + 1.738·*R*_C_ (0.91), and the average distance between cations A and B and fluorine (*R*_A–F_ + *R*_B-F_)/2(Å) = 1.13 + 1.1·*R*_C_ (0.98) and *R*_C-O_(Å) = 1.264 + 0.03·*R*_C_ (0.86). These formulae can predict the corresponding values for other fluorocarbonates. Figure 5 shows the cell volumes per formula unit calculated using functional PBE + D3 and those measured experimentally in [2,8,9]. It also illustrates the average distances between cations and fluorine atoms, as well as the predicted and experimental values for KMgCO_3_F (*Z* = 3), RbZnCO_3_F (*Z* = 2), RbPbCO_3_F, and CsPbCO_3_F (*Z* = 1), which were beyond the scope of this work. Figure 5 clearly demonstrates a sufficient agreement between the calculated, predicted, and experimental values.

### 3.2. Electron Structure

**Figure 6:**
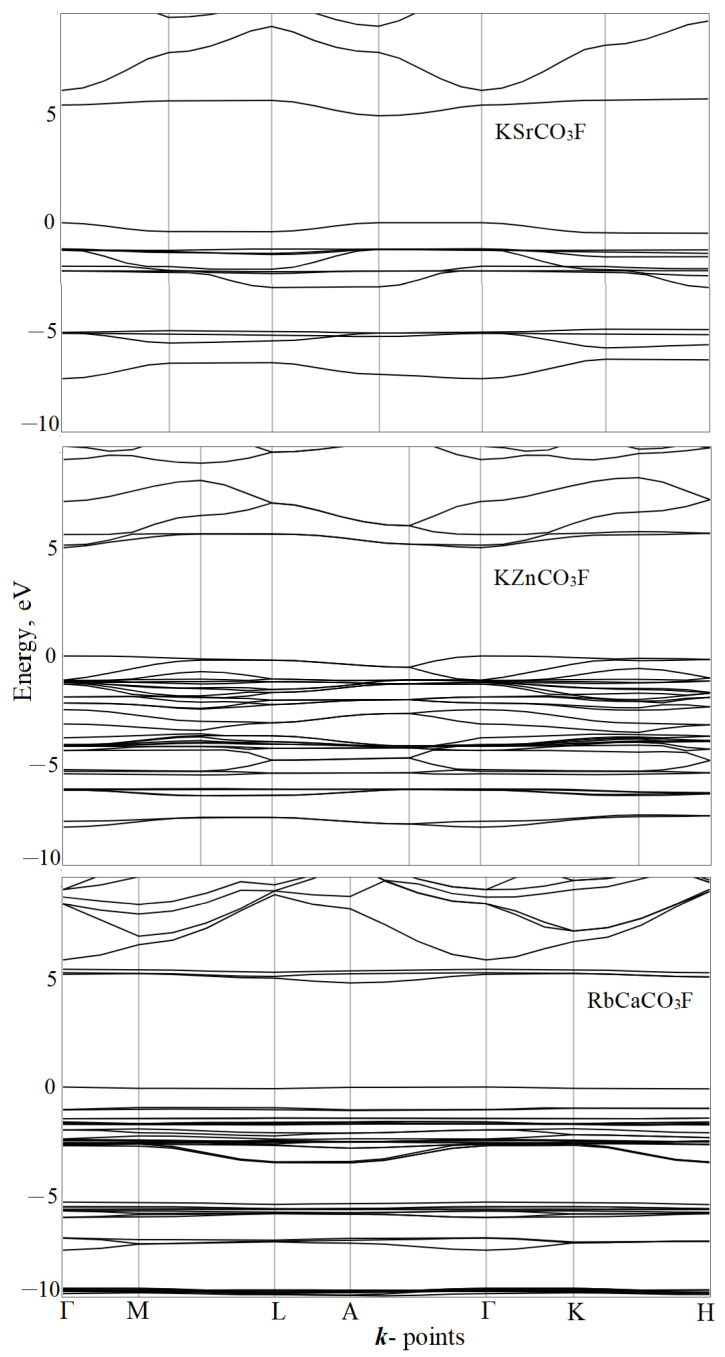
Band structures KSrCO_3_F (**top**), KZnCO_3_F (**center**), and RbCaCO_3_F (**bottom**).

The energy distribution of electrons in a crystal has a band structure that reflects the dependence of the electron energy on wave vector *E*(***k***). The relevant points of the Brillouin zone were Г(0,0,0), M(1/2,0,0), L(1/2,0,1/2), A(0,0,1/2), K(1/3,1/3,0), H(1/3,1/3,1/2). Figure 6 visualizes calculations for the band structures of typical representatives KSrCO_3_F, KZnCO_3_F, and RbCaCO_3_F.

The last full state is taken as the energy reference point. Therefore, the occupied (valence) states have negative energies, and the unoccupied (conduction bands) have positive ones. Here and below, we give PBE + D3 calculations by default. The valence bands are narrow in energy *E* and practically flat, depending on ***k***. This pattern is typical of crystals with an ionic chemical bond. The conduction bands have a greater dispersion than the valence bands. The energy distance between the top of the valence band and the bottom of the conduction band determines an important parameter we defined as the band gap *E_g_*. The zones are divided into direct and indirect. In direct zones, the electron transition occurs from an occupied to an unoccupied state, both belonging to the same wave vector ***k***. In indirect zones, they belong to different ones.

In the fluorocarbonate group I, the top of the valence band and the bottom of the conduction band occur at point A. The band gaps in this series are 5.18, 5.12, and 5.32 eV. The uppermost valence bands are 0.78, 0.50, and 0.02 eV wide. Each is separated from the previous band by a band gap of 0.6–0.7 eV. This band is formed of 98% *p*_xy_-oxygen. The bottom unoccupied bands are also separated from the subsequent ones by a band gap of 0.08, 0.40, and 1.67 eV. They are formed of about 60% *p_z_*- carbon and of about 39% oxygen. Only the following conduction bands are metallic in nature for 50–70% of *s*-potassium, rubidium, calcium, and strontium. The share of CO_3_ is 15–30%.

In KZnCO_3_F and RbCdCO_3_F, the top of the valence band and the bottom of the conduction band are at point Г. In this series, its width is 5.18 eV (3.17 in [8], 3.29 in [15]) and 5.27 eV (5.05 in [7], 5.35 in [8]). In KCdCO_3_F, the top of the valence band is on the line and the bottom of the conduction band is at point Г. The width of the indirect gap is 5.008 eV, while the straight one is 5.013 eV (5.11 in [7], 5.30 in [8]). The uppermost two valence bands are separated from the previous ones by a small band gap. Their widths are 0.58, 0.65, and 0.76 eV in KZnCO_3_F, KCdCO_3_F, and RbCdCO_3_F, respectively. They are formed of 80–85% p_xy_-oxygen, 12% of *d*-zinc, 7% of *d-*cadmium, and about 5% of *p_z_*-fluorine. The lower two unoccupied bands are formed of ~50% *p_z_*-carbon and ~35% of oxygen. They overlap with the subsequent ones, which are ~60% of *d-*metallic nature of zinc or cadmium.

In RbMgCO_3_F, the top of the valence band and the bottom of the conduction band are at point A. The band gap is 5.07 eV. A band gap of 0.75 eV separates the uppermost two valence bands from the previous ones and their width is 0.5 eV. They are formed of 98% of the *p_xy_*-states of O1 oxygen and have an anti-bonding character. The lower three unoccupied bands are also separated from the subsequent ones by a band gap of 0.4 eV. The lowest one is 87% formed of *p_z_*- states of carbon and oxygen from C2O_3_, and the other two are 92% formed of C1O_3_. Only the next conduction band has a metallic character at 29% *s*-rubidium and 37% magnesium.

The band structure of RbCaCO_3_F and CsCaCO_3_F fundamentally differs from that of the rubidium–magnesium fluorocarbonate. The top of the valence band is at point Г, while the bottom of the conduction band is at point A and its indirect band gap is 4.97 and 5.16 eV. The top three valence bands are 1.1 and 1.0 eV wide. They are separated by a band gap of 0.35 eV from the preceding ones. The top one of these bands is 95% formed of *p*_xy_-oxygen from C2O_3_, whereas the other two are 92% formed of C1O_3_. The bottom unoccupied three bands are separated from the subsequent bands by band gaps 0.44 and 0.11 eV. The two bottom ones are formed of *p_z_*- carbon (68%) and oxygen from C1O_3_, while the third one is formed of C2O_3_ (88%). The following conduction bands are metallic: s- rubidium—39%, cesium—73%, and calcium—26.8%, respectively.

**Figure 7:**
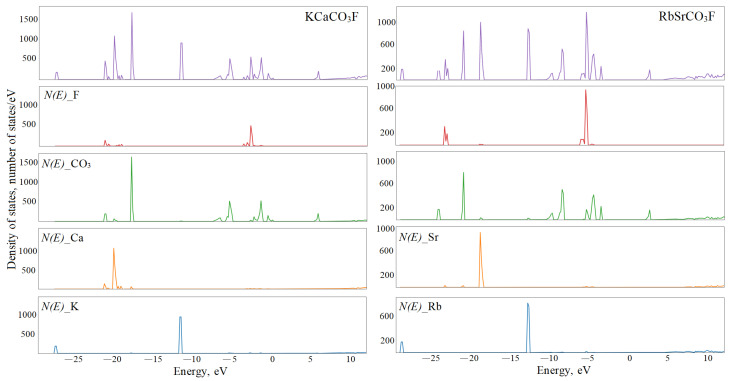

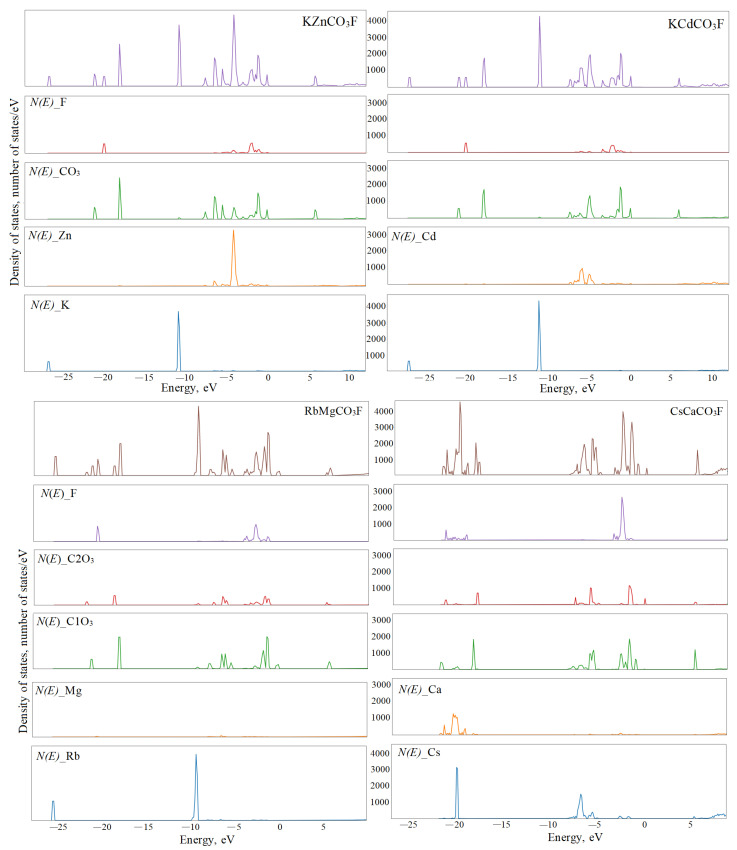
Total (**top**) and partial density of electronic states *N(E)* of fluorocarbonates.

The total and partial density distribution can characterize the nature of the electronic states (Figure 7). The energy position and width of bands *N(E)* reflect the hybridization degree.

The states of alkali metals hardly affect the formation of the upper valence region between −5 and 0 eV. In KCaCO_3_F and KSrCO_3_F, the K*_3s_*- and K*_3p_*-states of potassium are located in the region of −27.8, −28.4 and −11.9, −12.2 eV, respectively. Their respective widths are about 0.01 and 0.16 eV. Therefore, they participate very little in the chemical bond formation, hence the insignificant overlap population of their electron shells with other atoms. In KZnCO_3_F and KCdCO_3_F, the energy positions of bands K_3*s*_ (−27.1, −27.2 eV) and K_3*p*_ (−11.0, −11.3 eV) shift towards higher values. The width of the former remains insignificant, while that of the latter one rises to 0.28 and 0.17 eV, respectively. The energies of 4*s*- and 4*p*-states of rubidium shift towards lower values as the cation B radius increases: from −25.6 and −9.3 eV with widths of 0.05 and 0.71 eV in RbMgCO_3_F and −26.1 and −9.8 eV with widths of 0.02 and 0.36 eV in RbCaCO_3_F to −28.4 and −12.3 eV with widths of 0.00 and 0.15 eV in RbSrCO_3_F. The energy positions of Cs_5*s*_ and Cs_5*p*_ are −20.2 and −6.8 eV, and their widths are the most significant so far, namely 0.37 and 1.42 eV. This means that as the cation A radius increases at a fixed B, its states shift towards higher energies and the bandwidths increase.

Mg_2***s***_-, Mg_2*p*_-, Ca_3*s*_-, and Sr_4*s*_-lie in the energy region of the core states and are not marked in Figure 7. Ca_3*p*_- maintains its energy position as −19.6 eV, while its width increases from 1.33 eV in KCaCO_3_F to 1.57 eV in RbCaCO_3_F. These energies coincide with the region of states C_2*s*_-, O_2*s*_-, and F_2*s*_-. Therefore, their considerable width indicates mutual hybridization. The energies of Sr_4*s*_- states in KSrCO_3_F are at −15.3 eV with a width of 0.74 eV, while in RbSrCO_3_F they are at −15.6 eV with a width of 0.62 eV, which is higher in energy than the bands of hybrid anion *s*-states.

Zinc 3*d* states range from −5.5 to −4.0 eV with a maximal *N(E)* at −4.3 eV. They overlap with the region of C_2*p*_-O_2*p*_ hybrid states from below and F_2*p*_-O_2*p*_ states from above. Zinc states have a high degree of hybridization not only in this energy region but even in the upper valence states. For KCdCO_3_F, the band of cadmium 4*d* states ranges from –7.2 to –5.4 eV with maxima at −6.2 and −5.2 eV. The total share of cadmium for this energy range is 70%, oxygen—20%, and fluorine—5%. It is this bundle of energy bands that is responsible for the overlap population of Cd-F electron shells at 0.046 *e* and Cd-O at 0.057 *e*, which is approximately 70% of their total population. In RbCdCO_3_F, cadmium *d-*bands are located in the same energy region with a maximum of 6.3 eV. Anion–cation hybridization *p-d* is responsible for the overlap population of the Cd-O bond as 0.057 *e* and Cd-F as 0.037 *e*. The bottom hybrid zones with maxima at −6.7 and −7.5 eV provides 0.03 *e* of Cd-O overlap population. However, the upper bands with maxima at −5.1 eV are anti-bonding for Cd-O and bonding for C-O with 0.115 *e* of overlap population.

Structure *N(E)* of RbMgCO_3_F reflects the anion states since magnesium has almost no effect on its formation. The lowest bands with maxima at −22.1 and −21.4 eV are 0.74 eV wide. They are formed by hybridized *s-*states of carbon and oxygen, which are responsible for the C1-O1 overlap population of 0.106 *e* and C2-O2 of 0.101 *e*. Three bands at −20.8 eV are 0.12 eV wide. This group is formed by 95% of the fluorine *s-*states. The bands with *N(E)* maxima at −18.9 and −18.3 eV are 0.69 eV wide. They are formed by two-thirds by C1O_3_ *s-*states and by one-third by C2O_3_. They provide an overlap population of 0.178 *e* for C1-O1 and 0.16*e* for C2-O2. The energies of the *p-*states of oxygen and carbon (60% C1O_3_, 30% C2O_3_) range from −8.3 to −5.4 eV with *N(E)* maxima at −8.0, −6.6, −6.2, and −5.5 eV. They provide an overlap of 0.071 *e* for Mg-O1, 0.014 *e* for Mg-O2, 0.131 *e* for C1-O1, and 0.178 *e* for C-O2. The bands in the energy range from –4.2 to –3.5 eV are formed of the p*_z_*-states of fluorine (73%) and magnesium (6%). They contribute 0.037 *e* to overlap population Mg-F. These crystalline orbitals provide π-conjugation of the layers in the crystal by developing ···F-Mg-F···chains.

### 3.3. Effect of Pressure on Crystal Structure

**Figure 8:**
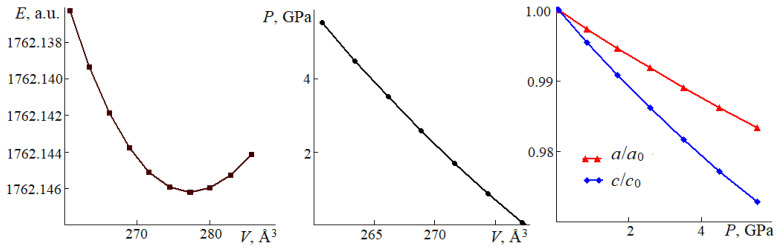
Total energy *E* vs. pressure *P* on cell volume *V*; ratio of lattice constants *a*/*a*_0_, *c*/*c*_0_ vs. pressure in RbMgCO_3_F. Solid lines denote approximation; figures represent the calculated data.

Pressure provides a good opportunity to study the interactions inside a crystal of structural elements. External voltage makes atoms leave their equilibrium positions. As a result, the bond lengths change in one way or another, depending on the chemical bonding strength. We used hydrostatic compression when the cell volume was reduced by a given value. After that, the structure parameters underwent complete optimization by calculating the total energy minimum. The resulting dependence *E(V)* was approximated by the third-order Birch–Murnaghan analytical equation of state. The distances–pressure dependence *R(P)* are described using compressibility modulus *K_R_* = *R*_0_/(*dR*/*dP*), where the derivative is calculated from a quadratic dependence. Figure 8 exemplifies dependencies *E*(*V*), *P*(*V*), *a*(*P*)/*a*_0_, and *c*(*P*)/*c*_0_. The equilibrium values of the crystal parameters (Table 1) are marked as zero. Table 2 shows the numerical values of EoS parameters and moduli obtained by functional PBE + D3.

For each group of fluorocarbonates, the cell volume per formula unit increases as *V*_0_/*Z*(Å^3^) = −59.27 + 120.83·*R*_C_ (0.99) following the increase in the average cation radius. The volume modulus decreases, which provides a reliable correlation for the entire *K*_0_(GPa) = 168.37 − 78.24·*R*_C_ (0.81). Fluorocarbonates have similar values of moduli *K*_0_. They are lower than those of calcite or dolomite and approach 65.24 GPa for aragonite [39]. RbMgCO_3_F and KZnCO_3_F with the smallest grade B cations have the maximal *K*_0_ values (minimal compressibility). KCdCO_3_F and RbMgCO_3_F have the maximal SGH coefficients. Their maximal volume modulus increase rate with *K*_1_ pressure is maximal. Their linear correlation coefficient SGH = −16.8 + 4.11·*K*_1_ is 0.70. The ion exceeds *D* for the dispersive energy per one formula unit *E*_disp_/*Z*, which is also at its maximum in these carbonates: −22.96 and −23.04 kJ/mol.

For each group of crystals, compressibility moduli *K_a_* along axis *a* and *K_c_* along axis *c* decrease as the average cation radius increases. However, group II is an exception: this rule does not work for module *K_c_* in RbCdCO_3_F. As for groups I and III *K_a_* > *K_c_*, this ratio increases in the former following the growing average cation radius and decreases in the latter. In group II, *K_a_* < *K_c_* and their ratio also decrease following the growing cation radius. In KZnCO_3_F, both directions along axes *a* and *c* also change with pressure.

Compressibility modulus *K*_A-O_ for the distance between metal A and oxygen O decreases in each group with an increase in the average cation radius, while the distances *R*_A-O_ grow. The linear dependency between them is stable, e.g., in group I: *K*_A-O_(GPa) = 425.8–97.92·*R*_A-O_. This pattern also exists for *R*_B-O_ distances in groups I and III. However, group II has a much higher compressibility modulus *K*_B-O_ than other fluorocarbonates with alkaline-earth metals. As mentioned above, Zn-O and Cd-O have high overlap populations. The fact that the region between the nuclei contains electrons prevents the distance from decreasing. This tendency is especially obvious in bond C-O, where compressibility modulus varies from 1000 to 1500 GPa, i.e., it is practically incompressible. In Table 2, group III has the average *K*_C-O_ value between two non-equivalent CO_3_ groups to illustrate the comparison. The compressibility modulus *K*_C1-O1_ for C1O_3_ in RbMgCO_3_F is 922 GPa. For *K*_C2-O2_, it is as high as 2042 GPa, which is almost 2.5 times bigger. On the contrary, in RbCaCO_3_F and CsCaCO_3_F, compressibility modulus *K*_C1-O1_ is 1753 (1572) GPa for C1O_3_ group and ≤871 (830) GPa for *K*_C2-O2_. This fact can be explained by the different values of distances C–O, which have already manifested themselves in the band structure.

The shortest distances between alkali metal atom A and fluorine atom *R*_A-F_ occur in plane *ab* (see Figure 1), and those between cations B and fluorine *R*_B-F_ occur along axis *c* (Figure 2). Under pressure, they change in proportion to the change in lattice constants *a* and *c*, respectively. For example, in KCaCO_3_F, it is *R*_A-F_(Å) ≈ 0.577·*a* and *R*_R-F_(Å) ≈ 0.577·*c*. Linear moduli have a similar relationship, which can be used to calculate them: *K*_A-F_(GPa) = −44.89 + 1.22·*K*_a_ (0.97), *K*_B-F_(GPa) = −9.95 + 1.05·*K*_c__._

### 3.4. Elastic Properties

Elastic properties determine how a certain material deforms or changes shape under applied forces. In microscopic theory, the elastic behavior of a crystal is related to the second derivative of the free energy with respect to reversible physical deformation. Thus, crystal elasticity is a very sensitive tool that can detect interactions and forces that occur between atoms and determine the structure and stability of crystals.

The elastic constant matrix *C_ij_* (*i, j* = 1, 2, 3) for hexagonal crystals contains five independent constants and *C*_66_ = (*C*_11_-*C*_12_)/2 (see Table 3 for PBE + D3 calculations).

Elastic constants determine the properties of mechanical stability, which look as follows in hexagonal crystals [40] *C*_44_ > 0, *C*_11_ > |*C*_12_| and $\left(C_{11}+C_{12}\right)\cdot C_{33}>2\cdot C_{13}^{2}$. Table 3 demonstrates that these conditions are satisfied for all crystals. Diagonal constants *C*_11_ and *C*_33_ indicate the response of the crystal to external stress along one axis, while *C*_44_ and *C*_66_ do the same for the plane shift. For groups I and II, *C*_33_ is greater than *C*_11_, which means that direction *c* is harder than *a(b)*. As *C*_12_ > *C*_13_, it means that if the same normal stress acts in direction *x*, the crystals in direction *z* shrink more than in direction *y*. Small *C*_44_ values indicate that shift deformation occurs more easily if stresses are applied to horizontal planes *ab*. Since *C*_66_ > *C*_44_, a shift in plane *ab* causes less response than in the perpendicular direction. C_44_ value can serve as an indicator to determine the cracking of a hexagonal crystal along axis *c*.

In fluorocarbonates, *C*_11_ decreases as the average cation radius increases. Group I has the following dependence: *C*_11_(GPa) = 306.0 − 137.8·*R*_C_. This downward trend in *C*_11_ indicates that the bond between ${\mathrm{CO}}_{3}^{2-}$ and cations becomes more ionic.

No experimental data for elastic constants are available; therefore, we have to compare their values obtained with different functionals. Constants *C*_11_ and *C*_33_ always satisfy the following condition: *C*_11,PBE + D3_ > *C*_11,B3LYP_ > *C*_11,PBE_, which means that the van der Waals interaction enhances the bond between BCO_3_ molecules and between layers. The shift constant, on the contrary, always satisfies *C*_44,B3LYP_ > *C*_44,PBE_ > *C*_44,PBE + D3_. Elastic constants *C_ij_* calculated by different functionals have a linear dependency. For example, for KSrCO_3_F, it looks like *C*_PBE_(GPa) = 1.38 + 0.90·*C*_PBE + D3_, *C*_B3LYP_(GPa) = 1.01 + 0.94·*C*_PBE + D3_; for KCdCO_3_F, it is *C*_PBE_(GPa) = 1.20 + 0.91·*C*_PBE + D3_, *C*_B3LYP_(GPa) = 0.97 + 0.92·*C*_PBE + D3_; and for RbMgCO_3_F, it is *C*_PBE_(GPa) = 1.23 + 0.92·*C*_PBE + D3_, *C*_B3LYP_(GPa) = 1.13 + 0.97·*C*_PBE + D3_.

Natural minerals are polycrystalline aggregates and represent a set of randomly oriented single crystals. The study of their mechanical properties is possible in two extreme cases: (1) any uniform deformation in a polycrystalline aggregate is equated to the value of external deformation; (2) uniform stress is equated to external stress. These two assumptions of uniform local strain and uniform local stress are known as the Voigt (*K*_V_, *G*_V_) [41] and Reuss (*K*_R_, *G*_R_) [42] approximations. These values can be obtained from the elastic constants using known formulae [43]. The Hill averaging method provides the best results [44]: volume and shift moduli can be determined as *K*_H_ = (*K*_V_ + *K*_R_)/2, *G*_H_ = (*G*_V_ + *G*_R_)/2. These values [45] make it possible to calculate the Young modulus *E*_H_ = 9*K*_H_*G*_H_/(3*K*_H_ + *G*_H_) and the Poisson ratio µ = (3*K*_H_ − 2*G*_H_)/(2(3*K*_H_ + *G*_H_)) (Table 4).

A crystal cannot physically exist outside *K*_V_/*K*_R_ < 1 and *G*_V_/*G*_R_ < 1. For all fluorocarbonates, these ratios are ≥1, but by a very small amount. The *K*_V_/*K*_R_ ratio is minimal for KZnCO_3_F (1.000) and KCaCO_3_F (1.0003); it is maximal for RbMgCO_3_F (1.034) and RbCdCO_3_F (1.0205). Other crystals have intermediate values. These ratios are included in the so-called universal anisotropy index [46] *A_U_* = 5·*G*_V_/*G*_R_ + *K*_V_/*K*_R_-6, which takes into account elastic anisotropy and is equally valid for all types of crystals (symmetries). The orthorhombic phase of carbon has the highest anisotropy, where *A_U_* is 397.3. Trigonal carbon has 284.0 [47]. Only RbCaCO_3_F (1.6) and KSrCO_3_F has *A_U_* ≥ 1 (1.18). It is at its lowest in CsCaCO_3_F (0.27) and RbCdCO_3_F (0.41).

**Figure 9:**
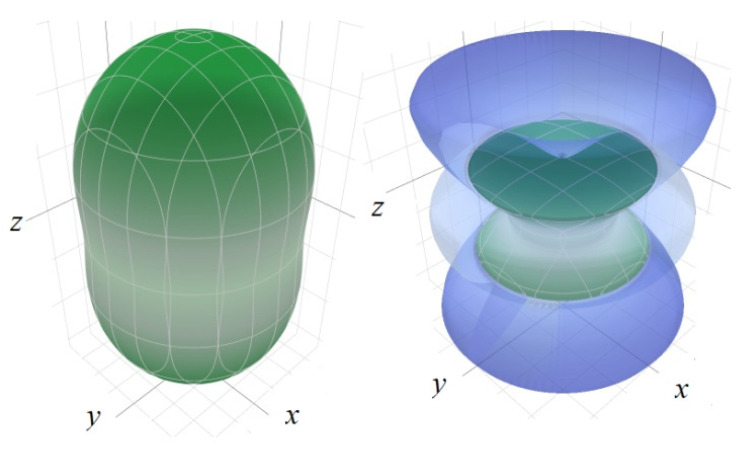
Anisotropy of linear compressibility for RbMgCO_3_F (**left**) and shift modulus for RbCaCO_3_F (**right**).

Anisotropy is an important property of any polycrystalline material. The anisotropy coefficient for each modulus can be calculated through its maximal and minimal values for each direction [48]. For linear compressibility (β = 1/*K*_H_), the direction *a(b)* has a minimal value of β_min_, and axis *c* has a maximal value of β_max_ (Figure 9). The maximal anisotropy coefficients for β belong to RbCdCO_3_F (1.75) and RbMgCO_3_F (1.71), while the minimal value belongs to KZnCO_3_F (1.00). The minimal value of shift modulus *G*_min_ in all fluorocarbonates is in the direction of axis *c* and the maximal value is in various combinations of *a(b)c*. The maximal anisotropy coefficient for the shift modulus belongs to RbCaCO_3_F (3.33): it is ≥ 2 everywhere except RbCdCO_3_F (1.82) and CsCaCO_3_F (1.67). The maximal Young modulus *E*_max_ is along axis *c.* Its maximal anisotropy coefficient belongs to RbCaCO_3_F (2.58) and RbSrCO_3_F (2.45), whereas the lowest value is in CsCaCO_3_F (1.44). The Poisson ratio also has anisotropy but it is impossible to indicate any single selected direction for all materials.

For each group of crystals, elastic moduli *K*_H_, *G*_H_, and *E*_H_ decrease as the average cation radius increases. For each modulus, its value is maximal in group III and minimal in group I. Fluorocarbonates with a small grade B cation radius have the maximal *K*_H_. The modulus has a reliable linear dependence on average cation radius *K*_H_(GPa) = 171.1–79.9·*R*_C_ (0.81).

Shift modulus *G* describes plastic deformation resistance and volume modulus *K* reflects fracture resistance. Therefore, the brittle or plastic behavior of solids can be predicted based on the simple empirical relationship that occurs between them. The critical value that separates them is 1.75. If *K*/*G* > 1.75, the material is plastic; otherwise, it is brittle. Table 4 demonstrates that all the fluorocarbonates in this research are plastic materials.

The Poisson ratio provides information about the characteristics of the bond strengths. For central forces in solids (ionic crystals), value μ = 0.25 (*G/K* = 0.6) is the bottom limit, while 0.5 (*G/K* = 0) is the top limit. At μ ≤ 0.25 (*G/K* > 0.6), the interatomic forces are non-central, which indicates that the bond has a covalent component. In fluorocarbonates, μ ≥ 0.28 indicates central forces and ionic bonding. The Poisson’s ratio and ratio (*G/K*) = 1.238–2.588 µ have a reliable linear dependency: group II fluorocarbonates with Zn and Cd and µ > 0.34 have chemical bonding with a greater covalent character than other fluorocarbonates. The same crystals also have greater plasticity, since for them *K*/*G* > 2.8.

The resulting set of elastic moduli can be used for semi-empirical estimates of some other physical properties of polycrystalline materials. For instance, acoustic velocities in a solid can be obtained from the volume modulus, shift modulus, and density ρ. In an anisotropic material, wave energy propagates in two modes, i.e., longitudinal and transverse. As a rule, the longitudinal mode is faster: particle oscillations move parallel to wave energy propagation. The transverse mode is slower: particle oscillations are perpendicular to wave energy propagation. Acoustic velocities of longitudinal (*v*_p_) and transverse (*v*_s_) waves can be theoretically defined as [49]: *v_P_* = ((*K*_H_ + 4*/*3*G*_H_)/ρ)^1*/*2^, *v_S_* = (*G*_H_/ρ)^1*/*2^. The highest velocities of the longitudinal and transverse waves belong to KCaCO_3_F and RbMgCO_3_F. In each group, they decrease following the increase in the average cation radius. For example, it is *v*_s_(km/s) = 11.74 − 6.22·*R*_C_, *v_P_*(km/s) = 22.10 − 11.77·*R*_C_ in group I.

### 3.5. Vibration Spectrum

The spectra of infrared light absorption (IRS) and Raman light scattering (RS) of fluorocarbonates are divided into two regions: (1) lattice vibrations that involve metal atoms, fluorine, translational, and rotational displacements of whole carbonate groups; (2) intramolecular vibrations of atoms of carbonate groups. Free ${\mathrm{CO}}_{3}^{2-}$ ion (symmetry D_3h_) has four main IR-active vibrational modes [50]: symmetric stretching *ν*1 (1100 cm^−1^), out-of-plane bending *ν*2 (800 cm^−1^), singular asymmetric stretching *ν*3 (1400 cm^−1^), and singular plane deformation mode ν4 (700 cm−1). The same types of vibrations are active in RS.

Table 5 illustrates a classification of optical modes according to irreducible representations of the symmetry groups of fluorocarbonates, the nature of vibrations as external (lattice) or internal, and their activity in IRS and RS. Table 5 contains no data for symmetries *A*_1u_ and *A*_2g_, which are inactive for the indicated spectra. Table 6 gives wave numbers of vibrational modes.

For group I fluorocarbonates of symmetry *P*-6*m*2, only one mode where polarization vectors transform according to the irreducible representation $A_{1}^{\prime }$ is active in RS. Its α*_xx_*, α*_yy_*, and α*_zz_* tensor components are nonzero. This vibration belongs to the intramolecular *v*1 type. As the number of carbonate groups increases, the total number of these vibrations for group II with symmetry *P*-6*c2* and group III with symmetry *P*-62*m* is 2 and 6, respectively. Doubly degenerate modes of symmetry $E^{\prime }$ are active both in IRS with the polarization of the electric field vector ***E***||***z*** and in RS with nonzero components α*_xx_*, α*_xy_*, and α*_yy_*. Group I fluorocarbonates have only five such modes: two internal modes *v*4 and *v*3 and three lattice modes. Crystals of symmetry *P*-62*m* and *Z* = 3 have only fourteen: six internal modes, three *v*4 modes, three *v*3 modes, and eight lattice modes.

**Figure 10:**
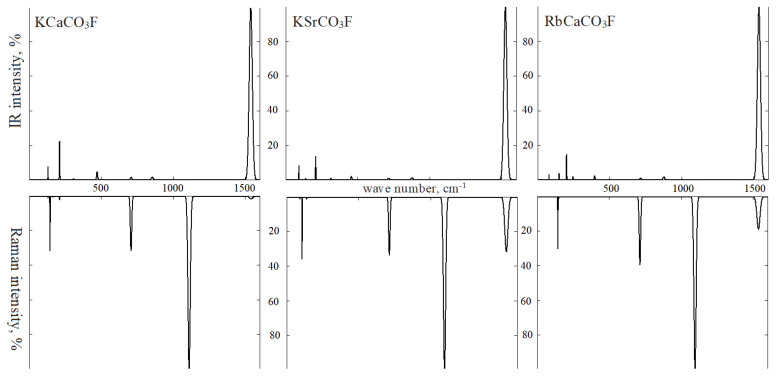
Infrared (**top**) and Raman (**bottom**) spectra: KCaCO_3_F (**left**), KSrCO_3_F (**center**), and RbSrCO_3_F (**right**).

Figure 10 shows the normal long-wave oscillations calculated using functional B3LYP, as well as the IRS absorption and RS of group I fluorocarbonates obtained by their Gaussian broadening. The intensities are calculated as a percentage for ease of comparison. In the IRS spectrum of each crystal, the most intense intramolecular vibration *v*3 is taken as 100%. Its intensity decreases in the series as 2207, 2161, and 1964 km/mol with a decrease in the average Born charge of *Z*_C_ cation. For all the carbonates in this study, the maximal intensity averaged over number *Z* of modes *v3* equals *I*(km/mol) = −2337 + 2727·*Z*_C_ (0.91). The dynamic charge of alkali cations *Z*_A_ correlates with their electronic charge *Z*_A_(|*e*|) = 1.85 − 0.83·*Q*_A_ (0.93), and this correlation is the same for *Z*_B_(|*e*|) = 2.83 − 0.445·*Q*_B_ (0.96). The intensity of line *v*1 of the symmetry $A_{1}^{\prime }$ was taken as 100% in RS.

In IRS, KCaCO_3_F, modes *v*4 of $\mathrm{symmetry}\;E^{\prime }$ and modes *v*2 of symmetry $A^{\prime \prime }$ have an intensity of ~1.5% and are almost invisible in the spectrum. RS is dominated by mode *v*1 with wave numbers that decrease in series I as 1110, 1096, and 1096 cm^−1^, as well as by modes *v*4 (~35% intensity) and modes *v*3 (2, 32, and 19% intensity), which makes it possible to identify fluorocarbonates.

Not only wave number and intensity can characterize lattice modes. In the case of carbonate ions, the squared oscillation amplitude (percentage) of an atom or group of atoms is another option. In IRS group I, lattice modes of symmetry $E^{\prime }$ with wave numbers 130, 91, and 78 cm^−1^ are formed by translational vibrations of cations A with CO_3_ anions and cations B with fluorine anions in opposite directions of plane *xy*. Alkali metal atoms provide the maximal contribution of 40–50% to the displacement vector amplitudes, while fluorine atoms are responsible for 50–40%. Modes with wave numbers 164, 138, and 148 cm^−1^ appear when A and F atoms (50%) shift in one direction, while B and CO_3_ (40%) atoms shift in the other direction. These are translational vibrations of two atomic planes relative to each other. The most intense modes at 210, 208, and 200 cm^−1^ are formed by translational shifts of cations A and B (46–10%) and anions CO_3_ (50–90%) and F in opposite directions. Symmetry vibrations $A^{\prime \prime }$ move cations and anions in opposite directions on axis *z*. Modes with low intensity and wave numbers 306, 313, and 245 cm^−1^ are formed by vibrations of atoms A, CO_3_ (65%) and B, F. Modes of high intensity at 471, 456, and 395 cm^−1^ are formed by vibrations of atoms B (27–10%) and fluorine (69–90%). In RS, librational oscillations of CO_3_ correspond to symmetry modes $E^{\prime \prime }$.

**Figure 11:**
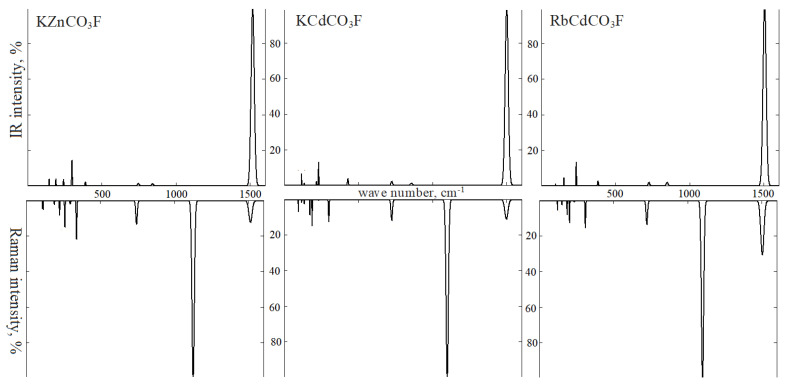
Infrared (**top**) and Raman (**bottom**) spectra: KZnCO_3_F (**left**), KCdCO_3_F (**center**), and RbCdCO_3_F (**right**).

For intramolecular vibrations of KCdCO_3_F in IRS (Figure 11), mode *v*3 of symmetry $E^{\prime }$ has a wave number of 1499 cm^−1^, its experimental value being1432 cm^−1^ [7]. In RbCdCO_3_F, it is 1505 cm^−1^ (experimental 1442 cm^−1^). Modes *v*4 with wave numbers ~720 cm^−1^ (730–680 cm^−1^) have a low intensity that starts at ~1%. Modes *v*2 of symmetry $A_{2}^{\prime \prime }$ with wave numbers 856 cm^−1^ (853 cm^−1^) and 847 cm^−1^ (843 cm^−1^) also have low intensity. In RS of cadmium fluorocarbonates, symmetry $A_{1}^{\prime }$ with wave numbers 1098 and 1100 cm^−1^ demonstrate the most intense vibration, while modes *v*4 and *v*3 have an intensity of ~7–10%.

For IRS of lattice vibrations in KCdCO_3_F, symmetry modes $E^{\prime }$ with wave numbers 231 cm^−1^ and 117 cm^−1^ prove to be the most intense ones (13%). In the first vibration, cadmium (3%) and CO_3_ (94%) atoms are displaced in opposite directions of plane *xy*. In the second vibration, Cd and CO_3_ atoms shift in the same direction, while potassium (40%) and fluorine (56%) atoms shift in the other direction (in-plane shifts). Symmetry mode $A_{2}^{\prime \prime }$ with wave number 429 cm^−1^ appears as potassium and fluorine atoms (~90%) shift in one direction *z*, while Cd and CO_3_ (~10%) shift in another direction. Symmetry mode $A_{2}^{\prime \prime }$ with wave number 217 cm^−1^ results from translational vibrations of cadmium atoms (15%) and UCO_3_ anions (80%).

As for group II fluorocarbonates, the most intense (~15–20%) lattice vibrations in RS are symmetry modes $A_{1}^{\prime }$ with wave numbers 340, 300, and 310 cm^−1^, as well as symmetry modes $E^{\prime \prime }$. This symmetry corresponds to immobile Zn (Cd) and C atoms, as well as potassium (rubidium) and fluorine atoms that vibrate in plane *xy* (~33%) and CO_3_ atoms that rotate in the direction of axis _z_ (~65%).

**Figure 12:**
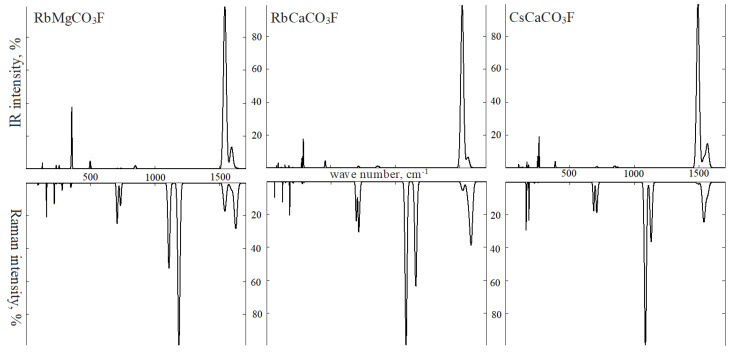
Infrared (**top**) and Raman (**bottom**) spectra: RbMgCO_3_F (**left**), RbCaCO_3_F (**center**), and CsCaCO_3_F (**right**).

In IRS of RbMgCO_3_F (Figure 12), mode *v3* with wave number 1539 cm^−1^ is the most intense one (5852 km/mol). It is followed by a mode with 13% intensity and a wave number of 1591 cm^−1^. The mode with 0.5% intensity at 1624 cm^−1^ comes last (experimental wave number 1495 and 1650 cm^−1^) [9]. Wave number gaps of 50 and 30 cm^−1^ prove that molecules interact. The contribution of the polarization vectors to mode I is 82% of C1O_3_ and 17% of C2O_3_. For mode II, it is 67% and 32%, respectively. For mode III, it is 50% of each. Three modes *v4* of symmetry $E^{\prime }$ have wave numbers of 705, 709, and 732 cm^−1^ (680 cm^−1^). Their intensity is ≤0.5%. The C2O_3_ group is responsible for 99% of the last mode. Two modes of type *v2* with intensities of ~2% have wave numbers of 842 and 846 cm^−1^ (890 cm^−1^). The third mode of this type with wave number 843 cm^−1^ belongs to symmetry $A_{1}^{\prime \prime }$ and is outside of the IRS scope. In BRS, modes v1 of symmetry $A_{1}^{\prime }$ with wave numbers 1184 cm^−1^ (100%) and 1107 cm^−1^ (52%) prove to be the most intense ones. They develop by 99.6% due to vibrations of oxygen atoms O1 and by 99.9% due to vibrations of oxygen atoms O2 along the C–O bond line.

The other two group III fluorocarbonates show changes in RS. RbMgCO_3_F has the second *v*1 maximum located to the left of the main one. However, in RbCaCO_3_F and CsCaCO_3_F it is on the right. The fact is that in fluorocarbonate with magnesium, bond length *R*_C1-O1_ is shorter than *R*_C2-O2_, whereas, in fluorocarbonate calcium, *R*_C1-O1_ is longer than *R*_C2-O2_. These structural features also lead to other changes in the spectrum. The positions of the wave numbers and mode *v3* intensities swap places. As a result, the central maximum changes at 1536 cm^−1^ in CsCaCO_3_F. The position of the maxima in band *v*4 also changes, while maintaining its width.

In lattice vibrations of RbMgCO_3_F, the IRS demonstrates an intense (37%) symmetry mode $E^{\prime }$ with wave number 354 cm^−1^. It depends on 60% of magnesium atom vibration, 23% on C1O_3_, and 16% on C2O_3_. Symmetry mode $A_{2}^{\prime \prime }$ with wave number 497 cm^−1^ (experimental 540 cm^−1^ [9]) and an intensity of 5% depends on 27% magnesium atom vibration and 71% on fluorine atoms. In RS, the most intense modes (10 and 6%) belong to symmetry $E^{\prime \prime }$ with wave numbers 159 cm^−1^ and 219 cm^−1^. Both are librational. The first is formed by vibrations of O1 oxygen atoms. The second involves magnesium and fluorine atoms in addition to O1 (36%) and O2 (30%).

The energy of zero-point vibrations $E_{\mathrm{ZP}}=\overset{N}{\underset{i=1}{\sum }}h\nu_{i}/2$ is an important energy characteristic of the phonon spectra of crystals. No accurate thermodynamic analysis of fluorocarbonate development is possible without it. The zero-point energy in terms of one formula unit has a correlation coefficient of 0.7. It decreases following the increase in the average ionic radius *E*_ZP_(kJ/mol) = 83.89 − 22.34·*R*_C_. The wave numbers of intramolecular vibrations also depend on the average radius of the cation. For mode *v4*, they increase in group I while decreasing (mean value) in groups II and III. As a result, their linear dependence has a correlation coefficient of at least 0.75 of *v*4(cm^−1^) = 883 − 123·*R*_C_. Out-of-plane vibration mode *v*2 (cm^−1^) = 690 − 123·*R*_C_ behaves approximately the same way. For the fully symmetric mode that dominates RS, it is *v*1(cm^−1^) = 1315 − 151·*R*_C_. These formulas can predict the IRS and RS of fluorocarbonates.

Tran et al. measured wave numbers of some intramolecular modes for RbMgCO_3_F in [9]. Their mean-square deviation for B3LYP is 4.7%, for PBE—5.9%, and for PBE + D3—4.6%. In the IRS of RbMgCO_3_F, functional PBE calculations of wave numbers for twenty active vibrations are connected by a linear dependency with the functional B3LYP as *v*_PBE_(cm^−1^) = −2.47 + 0.97·*v*_B3LYP_. For PBE + D3, it is *v*_PBE + D3_(cm^−1^) = 4.74 + 0.97·*v*_B3LYP_. For B3LYP, some frequency values are higher and some are lower than for PBE + D3, but they are always greater than PBE. For example, the lattice mode Mg-F of symmetry $A_{2}^{\prime \prime }$ for the functionals, B3LYP and PBE have a wave number of 497 cm^−1^, and for PBE + D3 its value is 537 cm^−1^. Therefore, intermolecular interaction agrees with the experimental value for this lattice mode. The situation is different for intramolecular modes, e.g., for KCaCO_3_F, it is as follows: *v*_PBE_(cm^1^) = −18.2 + 0.98·*v*_B3LYP_, *v*_PBE + D3_(cm^−1^) = −22.9 + 0.99·v_B3LYP_. Thus, the functionals PBE and PBE + D3 provide lower wave numbers than B3LYP. The ratio of zero-point energy obtained by functional B3LYP is 1.001 ÷ 1.036 times greater than by functional PBE and 1.00 ÷ 1.027 times greater than the functional PBE + D3.

## 4. Conclusions

Hexagonal fluorocarbonates ABCO3F (A: K, Rb, Cs; B: Mg, Ca, Sr, Zn, Cd) form structures of alternating layers ···B-CO_3_··· and ···A-F··· in planes *ab*, interconnected along axis *c* with infinite chains ···F-B-F···. As a result, cations form polyhedra AO*_n_*F_3_ and BO*_m_*F_2_. The average radius of cations *R*_C_ = (*R*_A_ + *R*_B_)/2 depends on coordination environment *n* + 3 and *m* + 2. The radius can establish linear dependences of structural and chemical bonding parameters. The resulting formulas can be used to predict the structural properties of other fluorocarbonates.

In this research, we studied the effect of pressure on the structure of fluorocarbonates. For volume compression moduli, the series dependence of the cation on the radius was *K*_0_(GPa) = 168.37 − 78.24·*R*_C_. The compressibility of metal–oxygen bonding for cations B was lower than that for alkali cations A, while bonds C-O were practically incompressible. For bonds A-F and B-F, we established the dependence on the linear compressibility moduli along axes *a* and *c* as *K*_A-F_(GPa) = −44.89 + 1.22·*K*_a_ and *K*_B-F_(GPa) = −9.95 + 1.05·*K*_c_, one of which can be used to calculate the other.

The elastic properties of monocrystalline and polycrystalline fluorocarbonates were anisotropic. Linear compressibility (1/*K*_H_) was minimal for direction *a*(*b*) and maximal along axis *c*. Shift modulus *G*_H_ (Young modulus *E*_H_) was minimal (maximal) along axis *c*. For each of the three groups of crystals with one, two, or three formula units Z, elastic moduli *K*_H_, *G*_H_, and *E*_H_ decreased as the average cation radius increased. We established a linear relationship between the Poisson ratio and ratio *G*_H_/*K*_H_: crystals with a larger proportion of covalent bonds were also more plastic.

The infrared absorption spectra for the most intense region with wave numbers 1490 ÷ 1620 cm^−1^ in the degenerate asymmetric extension modes of carbonate ion *ν*3 and the Raman scattering spectra in the most intense region of 1080 ÷ 1180 cm^−1^ in symmetric extension modes *ν*1 for fluorocarbonates with two crystallographically inequivalent carbonate groups had two maxima while the rest had one. The intensity of band *v*3*,* as well as the presence of one or two maxima *v1,* can be used to identify fluorocarbonates by their vibrational spectra.

The physicochemical change patterns for fluorocarbonates ABCO3F were established based on the cations. These patterns can serve as a basis for a programmed search for new NLO crystals with different cations. In addition, the new approach can be applied to the systematic analysis of physical characteristics of other halogen-carbonate families with different halogen/oxygen ratios in an anion sublattice.

## Figures and Tables

**Table 1:** Hexagonal cell parameters of fluorocarbonates calculated using functionals PBE + D3 and B3LYP and measured experimentally in [2,8,9] (Exp): parameters *a* and *c* and atomic coordinates.

Method	PBE + D3	B3LYP	Exp	PBE + D3	B3LYP	Exp	PBE + D3	B3LYP	Exp
	KCaCO_3_F			KSrCO_3_F			RbSrCO_3_F		
*a*, Å	5.0998	5.1434	5.0968	5.2274	5.2682	5.2598	5.2867	5.3287	5.3000
*c*, Å	4.4751	4.4951	4.4553	4.7074	4.7292	4.6956	4.8041	4.8246	4.7900
*x*_O_/*a*	0.1858	0.1880	0.1880	0.1889	0.1909	0.1916	0.1905	0.1926	0.1916
	KZnCO_3_F			KCdCO_3_F			RbCdCO_3_F		
*a*, Å	5.005	5.0591	5.0182	5.0846	5.1778	5.1349	5.2115	5.2907	5.2109
*c*, Å	8.3222	8.3473	8.355	8.7911	8.8933	8.846	9.0446	9.0514	9.0645
*x*_O_/*a*	0.4258	0.4250	0.4249	0.4122	0.4005	0.4062	0.4523	0.4493	0.4547
*y*_O_/*a*	0.3681	0.3597	03606	0.4611	0.4567	0.4530	0.3939	0.3816	0.3921
	RbMgCO_3_F			RbCaCO_3_F			CsCaCO_3_F		
*a*, Å	9.0247	9.0534	9.0160	9.2131	9.2712	9.1979	9.3239	9.4000	9.2999
*c*, Å	3.9321	3.9667	3.9403	4.4444	4.4785	4.4463	4.5628	4.6055	4.5400
*x*_Rb_/*a*	0.3861	0.3834	0.3825	0.2797	0.2813	0.2827	0.27996	0.2809	0.2798
*x*_Ca_/*a*	0.2779	0.2802	0.2812	0.3882	0.3863	0.3857	0.3870	0.3853	0.3857
*x*_F_/*a*	0.2748	0.2764	0.2788	0.3820	0.3804	0.3820	0.3848	0.3835	0.3828
*x*_O1_/*a*	0.1995	0.1984	0.2013	0.4855	0.4832	0.4842	0.3109	0.3124	0.3118
*y*_O1_/*a*	0.3151	0.3190	0.3167	0.2044	0.2054	0.2044	0.4834	0.4808	0.4816
*x*_O2_/*a*	0.1444	0.1430	0.1424	0.1413	0.1396	0.1387	0.1398	0.1380	0.1381

**Table 2:** Birch–Murnaghan equation of state: parameters *E*_0_ (a.u.), *V*_0_ (Å^3^), *K*_0_ (GPa), and *K*_1_; compressibility moduli of lattice constants *K_a_*, *K_c_* and interatomic distances *K*_A-O_, *K*_B-O_, *K*_C-O_ (GPa).

Crystal	*E*_0_	*V*_0_	*K*_0_	*K*_1_	*K*_a_	*K*_c_	*K*_A-O_	*K*_B-O_	*K*_C-O_
KCaCO_3_F	−1640.351	100.87	64.88	4.67	197	185	154	191	1181
KSrCO_3_F	−993.613	111.41	59.43	4.84	182	167	141	176	1319
RbSrCO_3_F	−418.049	116.24	56.10	4.91	174	157	135	168	1231
KZnCO_3_F	−5483.687	180.61	71.69	4.80	213	213	139	623	1179
KCdCO_3_F	−2261.171	200.31	60.16	5.12	166	211	124	4615	1119
RbCdCO_3_F	−1110.038	212.88	54.57	4.49	139	249	113	982	1046
RbMgCO_3_F	−1762.146	277.38	76.19	5.02	284	163	216	258	1482
RbCaCO_3_F	−3194.353	326.59	59.20	4.65	191	152	151	214	1312
CsCaCO_3_F	−3182.595	343.53	58.15	4.55	187	150	152	197	1202

**Table 3:** Elastic constants *C_ij_* (GPa) of fluorocarbonates.

Crystal	*C*_11_	*C*_12_	*C*_13_	*C*_33_	*C*_44_	*C*_66_
KCaCO_3_F	122.1	43.7	30.4	130.1	18.7	39.2
KSrCO_3_F	112.3	50.4	20.1	131.21	16.6	31.0
RbSrCO_3_F	107.0	41.9	23.9	112.9	17.7	32.6
KZnCO_3_F	114.0	54.8	46.8	122.2	16.4	29.61
KCdCO_3_F	90.8	48.6	35.1	124.8	15.3	21.1
RbCdCO_3_F	80.9	39.6	39.7	111.8	14.9	20.7
RbMgCO_3_F	152.8	67.5	37.1	122.3	21.5	42.7
RbCaCO_3_F	114.7	51.3	22.7	117.7	13.4	31.7
CsCaCO_3_F	109.9	43.4	29.0	105.5	23.3	33.2

**Table 4:** Elastic moduli (*K*_H_), shift moduli (*G*_H_), Young moduli (*E*_H_), Poisson ratio (µ), transverse (*v*_S_) and longitudinal (*v*_P_) acoustic wave velocities of polycrystalline fluorocarbonates.

Crystal	*K*_H_, GPa	*G*_H_, GPa	*E*_H_, GPa	µ	*v*_S_, km/s	*v*_P_, km/s
KCaCO_3_F	64.8	30.8	79.7	0.295	3.438	6.378
KSrCO_3_F	59.6	27.6	71.8	0.299	3.001	5.607
RbSrCO_3_F	56.2	27.3	70.4	0.291	2.754	5.073
KZnCO_3_F	71.9	24.5	66.0	0.347	2.707	5.591
KCdCO_3_F	60.2	21.4	57.5	0.341	2.361	4.805
RbCdCO_3_F	56.3	19.7	52.9	0.343	2.131	4.362
RbMgCO_3_F	77.7	33.8	88.5	0.31	3.164	6.032
RbCaCO_3_F	59.8	25.0	65.7	0.317	2.833	5.472
CsCaCO_3_F	58.5	30.1	77.1	0.28	2.871	5.197

**Table 5:** Number of optical vibrational modes for crystals with symmetry *P*-6*m*2/*P*-6*c2*/*P*-62*m*: lattice, CO_3_ internal, and total. Polarization activity is calculated for infrared absorption (IRS) and Raman scattering (RS).

Symmetry	IRS	RS	External	Internal	Total
$A_{1}^{\prime }$/$A_{1}^{\prime }$/$A_{1}^{\prime }$		α*_xx_*, α*_yy_*, α*_zz_*	0/1/4	1/1/2	1/2/6
$A^{\prime \prime }$/$A_{2}^{\prime \prime }$/$A_{2}^{\prime \prime }$	*E*||*z*		2/3/4	1/1/2	3/4/6
$E^{\prime }$/$E^{\prime }$/$E^{\prime }$	*E*||*xy*	α*_xx_*, α*_xy_*, α*_yy_*	3/5/8	2/4/6	5/9/14
$E^{\prime \prime }$/$E^{\prime \prime }$/$E^{\prime \prime }$		α*_xy_*, α*_yz_*	1/4/6	0/0/0	1/4/6

**Table 6:** Energy of zero-point vibrations *E*_ZP_/*Z* (kJ/mol) per formula unit; wave numbers of lattice (L) and intramolecular (*v*4*, v*2*, v*1*, v*3) vibrations (cm^−1^) of fluorocarbonates.

Symmetry	KCaCO_3_F	KsrCO_3_F	RbSrCO_3_F	KznCO_3_F	KCdCO_3_F	RbCdCO_3_F	RbMgCO_3_F	RbCaCO_3_F	CsCaCO_3_F
*E*_ZP_/*Z*	51.63	51.04	51.36	56.74	53.08	52.53	58.09	54.02	53.84
$L,\;E^{\prime }$	130	91	78	96	66	69	98, 126	80, 93	85, 103
$L,\;E^{\prime }$	164	138	148	190 145	135 117	152 95	234, 258 145	206, 145 113	166, 209 132
$L,\;E^{\prime }$	210	208	200	296 299	231 229	235 234	347, 274 354	276, 287 230	224, 250 260
$L,\;A_{2}^{\prime \prime }$	306	313	245	242	217	185	128, 182	117, 111	120, 158
$L,\;A_{2}^{\prime \prime }$	471	456	395	389	429	381	497, 256	457, 176	385, 178
$L,\;E^{\prime \prime }$				115 110	95 107	122 69	159, 90 98	123, 61 85	160, 111 79
$L,\;E^{\prime \prime }$	143	107	143	261 225	188 172	203 187	219, 280 496	178, 193 456	182, 201 385
$L,\;A_{1}^{\prime }$				340	300	310	214, 434 109, 86	299, 256 124, 84	260, 81 147, 227
*v* $4,\;E^{\prime }$	706	713	713	744 745	724 724	725 724	705, 732 709	694, 712 721	706, 683 714
*v* $2,\;A_{2}^{\prime \prime }$	853	876	875	841	856	847	846, 842	856, 869	845, 864
*v* $1,A_{1}^{\prime }$	1110	1096	1096	1126	1098	1100	1184, 1107	1079,1155	1084,1128
*v* $3,\;E^{\prime }$	1538	1526	1537	1517 1511	1499 1496	1501 1505	1539, 1591 1624	1517,1561 1583	1490, 1564 1536

## Data Availability

Data are available from the authors on request.
